# Bioprospecting of endophytic actinobacterium associated with *Aloe ferox* mill for antibacterial activity

**DOI:** 10.1186/s12906-022-03733-8

**Published:** 2022-10-03

**Authors:** Tsolanku Sidney Maliehe, Melusi Mbambo, Londeka Sibusisiwe Ngidi, Jabulani Siyabonga Emmanuel Shandu, Ofentse Jacob Pooe, Peter Masoko, Tlou Nelson Selepe

**Affiliations:** 1 Department of Biochemistry and Microbiology, Faculty of Science and Agriculture, University of Zululand, P/ Bag X1001, KwaDlangezwa, 3886 South Africa; 2 Department of Biochemistry, Microbiology and Biotechnology, University of Limpopo, Private bag X1106, Sovenga, 0727 South Africa; 3 School of Life Science, Discipline of Biochemistry, University of KwaZulu-Natal, Westville, 4000 South Africa; 4 Department of Water and Sanitation, University of Limpopo, Private bag X1106, Sovenga, 0727 South Africa

**Keywords:** Aloe, Endophytes, Secondary metabolites, Antibacterial activity, *Streptomyces olivaceus* CP016795

## Abstract

**Background:**

The emergence of drug resistance among pathogens has resulted in renewed interest in bioprospecting for natural microbial products.

**Methods:**

This study aimed to bioprospecting endophytic actinobacterium associated with *Aloe ferox* Mill for its antibacterial activity. Endophytic actinomycetes were isolated from the gel of *A. ferox* Mill by surface sterilization technique using actinomycete isolation agar. The isolate with a promising antibacterial activity was identified using 16S rRNA sequence analysis. The minimum inhibitory concentration (MIC) of the extract was assessed by the micro-dilution method and its effect on the respiratory chain dehydrogenase (RCD) activity was ascertained by the iodonitrotetrazolium chloride (INT) assay. Fourier transform-infrared spectrophotometer (FTIR) and gas chromatography-mass spectrophotometry (GC-MS) were employed to identify functional groups and the chemical constituents, respectively.

**Results:**

The actinobacterium was found to be *Streptomyces olivaceus* CP016795.1. Its extract displayed noteworthy antibacterial activity (MIC ≤1 mg/mL) against *Staphylococcus aureus* (ATCC 25925), *Bacillus cereus* (ATCC 10102), and *Escherichia coli* (ATCC 25922); and showed an inhibitory effect on the RCD activity. FTIR spectrum displayed hydroxyl, amine, and aromatic groups, and the GC–MS revealed 5-Hydroxymethylfurfural as the main constituent (19.47%).

**Conclusions:**

*S. olivaceus* CP016795.1 can serve as a potential source of effective antibacterial compounds.

**Supplementary Information:**

The online version contains supplementary material available at 10.1186/s12906-022-03733-8.

## Introduction

There is a growing concern worldwide concerning the ever-increasing incidence of antimicrobial resistance and dissemination of antimicrobial resistance genes amongst pathogens. The occurrence of drug resistance among pathogens limits the utilization of the currently available antimicrobial agents [1]. Antimicrobial resistance is a result of the presence of antimicrobial resistance genes that are transferred among microbial strains mainly by a horizontal gene transfer mechanism [2]. The main mechanisms of antimicrobial resistance are the innate hydrolysing activities of enzymes, the activities of an efflux pump that pump-out antimicrobials from cells, the use of DE novo metabolic pathways which provides altered cell walls that do not possess active binding sites for antimicrobials, formation of biofilm, quorum sensing and a plethora of mutations that interfere with antimicrobials to target sites [3]. Infectious diseases cause over 4.8% of mortality rate globally, per year and an economic loss of more than USD 100 trillion per year [4]. Thus, the discovery and development of alternative strategies to treat infections is the need of the day.

Bioprospection of endophytes is considered a novel frontier for the discovery of effective and safe compounds [5]. Endophytes colonize inter- or intracellular tissues without exhibiting pathogenicity [6]. Only 1% of the total microbial populations linked to plants are explored so far [7]. Moreover, approximately 70% of the endophytes that have been isolated and identified are fungal and the remaining portion is bacteria, mainly belonging to the four phyla: Firmicutes, Proteobacteria, Bacteroidetes, and Actinobacteria [8]. Actinomycetes are filamentous Gram-positive microorganisms with high G + C, belonging to the phylum Actinobacteria [9]. They are generally accepted as the asenals of bioactive compounds. More than 23,000 bioactive compounds have been synthesized by actinomycetes and they account for 45% of all active microbial metabolites. Among actinomycetes, streptomycetes are the most active compound producers and have provided more than 7600 of the currently known compounds [10]. These compounds have demonstrated potential applicability in medicine, agriculture, and industry [11]. Actinomycetes are ubiquitous in most plants; however, most have been isolated from soil and marine environments and not from the inner plant tissues [12–14]. Thus, endophytic actinomycetes represent an untapped niche of novel bioactive metabolites. Moreover, endophytic actinomycetes produce an array of compounds with novel skeletons-chemical structures [15].

*Aloe forex* Mill, commonly known as bitter aloe or Cape aloe is a species of flowering medicinal plant belonging to the family *Asphodelaceae*. The plant is commonly used as a source of antioxidant, antimicrobial, laxative, antidiabtic, antiinflammatory and antiarthritis agents [16–19]. Its leaves are heterogeneous and are divided into three major parts, namely: (i) the outer green epidermis, primarily consisting of structural components; (ii) the outer pulp region below the epidermis, consisting of vascular bundles where the bitter latex is derived; and (iii) the inner leaf pulp, consisting of aloe gel containing parenchyma cells. Regarding the different compositions of the leaf portions, they are also likely to have distinct classes of bioactive compounds, which are believed to contribute to the different biological properties [17]. Moreover, the gel possesses a high moisture content and various nutrients that support microbial growth and diversity. Although literature records the therapeutic value of *A. ferox* Mill [18, 19], there is negligence in terms of the importance of its endophytes in medicine*.*

This study aimed to isolate and identify an antibiotic-producing endophytic actinomycete from the gel of *A. ferox* Mill using conventional and molecular techniques. The antibacterial potential of its extract was assessed by a micro-dilution method. Moreover, the antibacterial mechanism was investigated by evaluating the effect of the extract on the respiratory chain dehydrogenase using an iodonitrotetrazolium chloride (INT) assay. Lastly, the functional groups and chemical composition of the extracted secondary metabolites were identified by Fourier transform-infrared spectrophotometer (FTIR) and standard biochemical methods, and gas chromatography-mass spectrometry.

## Materials and methods

### Plant selection and sampling

*A. ferox* Mill used in this study was selected based on its history of use in South African traditional medicine. Fresh leaves of *A. ferox*, were obtained in March 2021 at the University of Zululand, South Africa (28 °45 ′S31 °54 ′E). The plant was identified by Prof. Ntula at the Department of Botany, University of Zululand, South Africa. The voucher specimen for *A. ferox* species, voucher number TNS1 was prepared and deposited in the University of Zululand Herbarium [ZULU], which is available mainly to researchers. Ethical approval to collect the plant was obtained from the research ethical committee at the University of Zululand (UZREC 171110–030 PGM 2021/56). The use of the plant extract in this study complies with the international guidelinesThe leaves were washed with tap water to remove soil particles, air-dried to remove any moisture, and then packaged into a sterile collecting bag. Thereafter, the sample was transported to the Microbiology laboratory, where isolation of endophytic actinomycete procedure was instituted.

### Tissue preparation

Endophytic actinomycetes were isolated from the gel of *A. ferox* using a surface sterilization procedure as described by Nxumalo et al. [20]. Briefly, the leaves (*n* = 6) were removed from the sampling bag and washed with sterilised distilled water to remove debris. They were placed under a laminar airflow cabinet to dry and each leaf was cut into pieces of 3 cm. Four fragments from each leaf, taken randomly from different positions, were selected and dipped in 75% ethanol for 5 minutes followed by dipping into 2% sodium hypochlorite solution for 4 minutes. They were further dipped in 10% sodium bicarbonate for 3 minutes and subsequently washed six times using autoclaved distilled water. Thereafter, the fragments were dried using sterile paper towels. The gel fillets were extracted by removing the green rinds with a sterile knife, aseptically, washed in sterile distilled water to remove the latex and then ground by pestle and mortar. The ground tissue extracts were serially diluted in sterile saline solution (0.9% NaCl). The effectiveness of the sterilization procedure was assessed by plating 100 μL of the final sterile water rinse onto nutrient agar (NA) and actinomycete isolation agar with glycerol (AIAG) plates. The plates were incubated at 28 °C for 240 hours and observed for microbial growth.

### Isolation of endophytic actinomycetes

AIAG was used for the isolation of endophytic actinomycetes. About 100 μL of the undiluted and diluted samples were pipetted on the AIAG plates and spread evenly using a sterilised glass spreader. Thereafter, the plates were incubated at 28 °C for 240 hours. The colonies were counted as colony forming units per gram (CFU/g) and expressed as population density. The colonies were selected based on divergence in morphology, size, and color [21]. The isolates were sub-cultured twice on AIAG agar.

### Screening of production of antibaterial compounds

#### Primary screening of production of antibacterial compounds

The primary screening for the inhibitory action of the isolates was carried out using a modified cross streak method [CSM] using the production medium (yeast extract, 3 g; peptone, 3 g; casein, 3 g; starch, 8 g; K_2_HPO, 0.5 g; MgSO_4_7H_2_O, 0.5 g; NaCl, 2 g; agar, 15 g and pH 7.0). The above-mentioned composition was done in a Litre of the sterilised distilled water. The test bacteria - *Staphylococcus aureus* (ATCC 25925) and *Escherichia coli* (ATCC 25922) were streaked at the right angles to the line of the isolates and incubated for 24 hours at 37 °C. The inhibition of the test bacteria was measured in millimeters [22].

#### Secondary screening of production of antibacterial compounds

Isolates with promising antibiotic production were further selected for secondary screening based on their inhibition against *S. aureus* (ATCC 25925) and *E. coli* (ATCC 25922) using the agar well diffusion method. Isolates were inoculated into a sterile conical flask of 100 mL that contains the production medium (30 mL) and incubated for 10 days at 28 °C in a shaking incubator at 150 rpm. Thereafter, the culture broth was centrifuged at 5000 rpm for 30 minutes. The centrifugation procedure was repeated three times to ensure that the supernatant is cell-free before assessment of antibacterial activity using the agar well diffusion method. Briefly, the test bacterial inoculums- *S. aureus* (ATCC 25925) and *E. coli* (ATCC 25922) at the exponential growth stage were adjusted to 10^6^ CFU/mL. Thereafter, the lawns of the two test bacteria were made on Muller Hinton agar plates, followed by the construction of agar wells (6 mm diameter). The cell-free supernatant (100 μL) was transferred into the wells and incubated at 37 °C, overnight. The plates with un-inoculated supernatant served as controls. Growth inhibition zones were observed and recorded in milliliters [23].

### Identification of the endophytic actinobacterium

Due to financial limitations, only the most promising antibiotic-producing isolate-ISO1, was sent to Inqaba Biotechnical Industries (Pty) Ltd., in South Africa to be identified using 16S rRNA gene amplification and sequencing analysis. The genomic DNA was extracted from pure ISO1 isolate using the ZR Fungal/Bacterial DNA Kit™ (Zymo Research, Catalogue No. D6005) according to the manufacturer’s protocol. The 16S rRNA gene was amplified using primers (16S-27F (5′AGAGTTTGATCMTGGCTCAG-3′) and 16S-1492R (5′- CGGT TACCTTGTTACGACTT-3′) and DreamTaq™ DNA polymerase. Polymerase chain reaction (PCR) products were obtained through gel extraction; purified and sequenced in the forward and reverse directions. The purified sequencing fragments were run on the ABI 3500xl Genetic Analyzer (Applied Biosystems, ThermoFisher Scientific). CLC Bio Main Workbench v7.6 was used to evaluate the files generated by the ABI 3500XL Genetic Analyzer. The endophytic actinomycete ISO1 isolate was identified based on the similarity of the amplified sequence with those found in the National Centre for Biotechnology Information (NCBI) database using the Basic Local Alignment Search Tool (BLAST) [24].

### Biosynthetic gene clusters of the endophytic actinobacterium

The secondary metabolite biosynthetic gene clusters of the isolate were predicted using antibiotics and Secondary Metabolite Analysis Shell (anti-SMASH). Briefly, the accession number of the isolate was submitted to anti-SMASH. The produced information regarding the type of cluster, most similar known cluster, and percentage similarity was investigated [25].

### Solvent extraction of the secondary metabolites

A loopful of IS01 was inoculated into a sterile conical flask (100 mL) containing the production medium (50 mL) and incubated for 48 hours at 37 °C in a shaking incubator at 150 rpm. The resulting seed culture was adjusted to McFarland standard (1 × 10^6^ CFU/mL) and about 200 μL was then transferred into a flask (1000 mL) containing 500 mL of the same production medium. It was then incubated for 10 days at 28 °C under agitation at 150 rpm. The culture broth was centrifuged three times at 5000 rpm for 30 minutes. Thereafter, a three-solvent system comprising chloroform, ethanol, and ethyl acetate was used for the extraction of the secondary metabolites from the culture broth, separately. A ratio of 1:1 for solvent and broth was used for the extraction procedure. The extractable secondary metabolites were obtained from the solvent phase by evaporating the solvent under a laminar airflow cabinet. The obtained three extracts were combined and dissolved in 10% dimethyl sulphoxide (DMSO) at a concentration of 2 mg/mL and kept at 4 °C [20].

### Determination of antibacterial activity

#### Minimum inhibitory concentration (MIC) of the extract

The extract was subjected to analysis for its minimum inhibitory concentration (MIC) using the 96 well microdilution method as per the Clinical and Laboratory Standards Institute [26]. The extract solution in 10% DMSO was serially diluted with MH broth followed by pipetting of 100 μL of the fresh selected bacterial suspension (*Staphylococcus aureus* (ATCC 25925), *Bacillus cereus* (ATCC 10102), *Pseudomonas aeruginosa* (ATCC 27853) and *Escherichia coli* (ATCC 25922)); at a density of 1 × 10^6^ CFU/mL. Ten percent of DMSO served as negative control while ciprofloxacin (20 μg/mL) was used as a positive control. The plate was sealed and incubated at 37 °C for 24 hours. Afterward, 40 μL of 0.2 mg/mL of iodonitrotetrazodium violet (INT) solution was added to each well and re-incubated at 37 °C for 30 minutes. The MIC of the extract was taken as the lowest concentration of the extract that inhibited bacterial growth.

#### Minimum bactericidal concentration (MBC) of the extract

The MBC was evaluated by withdrawing 20 μL of bacterial suspensions from the wells that showed no growth during the evaluation of MIC. The suspensions were pipetted into 50 μL of NB in a sterile 96 well plate. The plate was incubated at 37 °C, overnight. Thereafter, 40 μL of INT was poured and the plate was re-incubated at 37 °C for 30 minutes. The lowest concentration that displayed no bacterial growth was identified as the MBC of the extract [27].

### Bactericidal and bacteriostatic effects of the extract

The ratio of MBC/MIC was calculated to characterize the antibacterial activity of the extract. When the ratio of MBC/MIC was ≤4, the extract was considered to be bactericidal and when the ratio was > 4, it was defined as bacteriostatic.

### Determination of the respiratory chain dehydrogenase activity

The effect of the extract on the respiratory chain dehydrogenase activity of the test bacteria was evaluated using the iodonitrotetrazolium chloride (INT) method. The bacteria were cultured on NB, incubated overnight at 37 °C, and adjusted to 10^6^ CFU/mL. Thereafter, 1 mL of bacterial suspensions was added into the sterile test tube, followed by 2 mL of 0.05 mol/L Tris-HCl buffer (pH = 8.6), 2 mL of 0.1 mol/L glucose solution, and 2 mL of 1 mg/mL triphenyl formazan solution. After mixing, the extract (MIC and MBC) was added and incubated at 37 °C for 6 hours. Two drops of concentrated sulphuric acid (H_2_SO_4_) were pipetted into each test tube to stop the reaction. Thereafter, 5 mL of n-butyl ethanol was used to extract the products. The upper organic phase was centrifuged at 5000 rpm for 15 minutes. The optical density at 490 nm was measured using n-butyl ethanol as a blank. The cells that were boiled for 30 minutes to inactivate their enzymes served as the negative control, while the positive control was the cells whose enzymes were maintained natively active by not boiling [28].

### Characterisation of the bacterial extract

#### Evaluation of functional groups of the extract

The Fourier transform-infrared spectrophotometer (FTIR) (PerkinElmer UATR TWO, 2000; Germany) was used to identify the functional groups of the extract. Briefly, the extract was firstly ground with KBr at 25 °C, pressed into a pellet, and then analysed at a wavenumber ranging from 4000 to 400 cm^− 1^ [29].

### Extract’s chemical constituents

The standard methods were used to screen different classes of compounds within the crude extract. The compounds that were screened included phenols, flavonoids, quinones, coumarins, anthraquinone and saponins. The visual observations of the reactions’ colouration properties or the precipitate formations were used to evaluate the presence or absence of the distinct compounds [30, 31].

### Identification of volatile secondary metabolites

The analysis of the volatile secondary metabolites was done using gas chromatography-mass spectrometry (THERMO Gas Chromatography TRACE ULTRA VER: 5.0.). Briefly, the flow rate of the helium gas was fixed to 1 mL per min, with a split ratio of 1:50. The injector temperature was set to 250 °C with the detector temperature adjusted to 280 °C. The temperature of the column was retained at 40 °C for 1 min followed by linear programming increasing the temperature from 40 to 120 °C. Two microlitres of the extract were injected for analysis. The mass spectra programmed in the scan mode were 70 eV in the range of 50–50 m/z [3].

### Statistical analysis

All experiments were done in triplicate and data was expressed as mean ± standard deviation. The statistical analyses were performed by one-way analysis of variance and were considered to be significantly different at *p* < 0.05.

## Results

### Surface sterilisation and isolation

AIAG plates that were inoculated with the gel samples from the leaves of *A. ferox* Mill displayed morphologically different colonies. There were white, pink, and pale yellow rough powdered colonies of 11 different varieties that were selected. The control plates did not show any bacterial or fungal growth after 48 hours.

### Screening for antibiotic production potential

The initial screening was done to evaluate the potential antibiotic production by the isolates against *S. aureus* (ATCC 25925) and *E. coli* (ATCC 25922), representing two classes of bacteria (Gram-positive and Gram-negative bacteria, respectively), and the results are shown in Table 1. Isolate ISO1, ISO2 and ISO3 demonstrated antibacterial activity against the tested pathogens in both primary and secondary screening procedures (Table 1). However, the activity was more profound with isolate ISO1 than with the other two isolates (ISO2 and ISO3).

**Table 1:** Screening of isolates for production of antibacterial agent

Isolates	Zones of inhibition (mm)				Bacterial name
	Primary screening		Secondary screening		
	***S. aureus***	***E. coli***	***S. aureus***	***E. coli***	
ISO1	+++	+++	+++	++	*S. olivaceus* CP016795.1
ISO2	+++	+++	++	+	
ISO3	+++	++	+++	+	
ISO4R	+++	+	–	–	
ISO5T	++	+	+	–	
ISO6D	++	+	+	–	
ISO7H	++	+	+	–	
ISO8B	+	–	–	–	
ISO9Y	+	+	–	–	
ISO10I	+	–	–	–	
ISO11C	+	–	–	–	

Inhibition zone diameter index: + (x ≤ 9 mm) weak activity, ++ (10–20 mm) moderate activity, +++ (x ≥ 21 mm) strong activity and - denotes no activity

### Identification of the isolate of interest

Molecular characterization of the endophytic actinomycete of interest (ISO1) revealed that it exhibits 99% similarity to *Streptomyces olivaceus* when compared to the NCBI database using the BLAST program. It was then recorded as *Streptomyces olivaceus* with accession number CP016795.1 (Table 1).

### Biosynthetic secondary metabolite gene clusters

*S. olivaceus* CP016795.1 was shown to possess 33 biosynthetic gene clusters that comprise 3 NRPS, 5 PKS, 7 hybrid gene clusters, 5 terpene, 3 siderophore, and 10 other biosynthetic gene clusters (Table 2). Approximately 48% of the revealed biosynthetic gene clusters showed more or equal to 50% (50 ≤ x) percentage similarity. The actinobacterium was also predicted to synthesise germicidin, ectoine, albaflavenone, phenalinolactone A, hopene, and geosmin with above 90% similarity to the gene clusters responsible for the production of these metabolites.

**Table 2:** The biosynthetic gene clusters associated with the secondary metabolites production by *S. olivaceus* CP016795.1

Regions	Type of Clusters	Most Similar Known Cluster	Similarity
1	NRPS-like, T3PKS, NRPS, T1PKS	Totopotensamide A/ totopotensamide B	82%
2	Terpene		
3	T3PKS	Germicidin	100%
4	Indole	5-isoprenylindole-3-carboxylate β-D-glycosyl ester	23%
5	Terpene	Carotenoid	54%
6	Amglyccycl	β-D-galactosylvalidoxylamine-A	22%
7	T3PKS	Herboxidiene	8%
8	NRPS	Rimosamide	21%
9	Ectoine	Ectoine	100%
10	Melanin	Melanin	60%
11	Lassopeptide	SSV-2083	36%
12	Siderophore	Desferrioxamin B/ desferrioxamin E	83%
13	LAP, thiopeptide	Diazepinomicin	7%
14	Lanthipeptide-class-ii	SBI-06990 A1/ SBI-06989 A2	50%
15	NRPS-like, NRPS, T1PKS	Azinomycin B	55%
16	Amglyccycl		
17	Batalactone	Julichrome Q3–3/ julichrome Q3–5	22%
18	Terpene	Albaflavenone	100%
19	T2PKS	Spore pigment	66%
20	Siderophore		
21	NRPS, NRPS-like, T1PKS	Auroramycin	7%
22	NRPS	Telomycin	29%
23	T1PKS	Xiamycin A	72%
24	RiPP-like		
25	Terpene	Geosmin	100%
26	Siderophore		
27	PSK-like, NRPS-like, terpene, NRPS, nucleoside	Phenalinolactone A	94%
28	Terpene, NRPS	Hopene	92%
29	NRPS, T1PKS, NRPS-like	Divergolide A/ divergolide B/ divergolide C/ divergolide D	100%
30	Terpene	Versipelostatin	5%
31	RiPP-like	Informatipeptin	42%
32	NRPS	Coelichelin	100%
33	T1PKS	Lobosamide A, lobosamide B, lobosamide C	19%

T3PKS, T1PKS, and LAP are abbreviations for type III polyketide synthase, type I polyketide synthases, and Linear azol(in)e-containing peptide, respectively

### The yield of the extract

Based on the profound antibacterial activity observed during the secondary screening of the antibiotic production, solvent extraction was used to obtain the crude secondary metabolites from the culture broth. ISO1- *S. olivaceus* CP016795.1 yielded 1.47 g/500 mL of the extract.

### Antibacterial activity of the extract

#### Inhibitory effect of the extract

The extract displayed broad-spectrum activity against the tested bacterial strains. The MIC values ranged from 0.05 to 2 mg/mL (Table 3). The inhibitory effect of the extract was more pronounced against the Gram-positive bacterial strains, *S. aureus* (ATCC 25925) and *B. cereus* (ATCC 10102), with the MIC value of 0.05 mg/mL on both. The Gram-negative strains, *P. aeruginosa* (ATCC 27853) and *E. coli* (ATCC 25922), had the MIC values of 2 and 1 mg/mL, respectively. However, the extract only did not display a noteworthy activity (MIC ≤1 mg/mL) against *P. aeruginosa* (ATCC 27853).

**Table 3:** MIC, MBC and MBC/MIC ratio values of the bacterial extract against the selected strains

Bacteria	Extract			Ciprofloxacin		
	MIC (mg/mL)	MBC (mg/mL)	MBC/MIC	MIC (μg/mL)	MBC (μg/mL)	MBC/MIC
*S. aureus* (ATCC 25925)	0.05	2	4	0.015	0.031	2
*B. cereus* (ATCC 10102)	0.05	2	4	0.015	0.06	4
*P. aeruginosa* (ATCC 27853)	2	> 2	–	0.06	0.24	4
*E. coli* (ATCC 25922)	1	> 2	–	0.015	0.031	2

### MBC and MBC/MIC ratios of the extract

The MBC assay was assessed against all the test bacterial strains. The most potent activity was displayed against *S. aureus* (ATCC 25925) and *B. cereus* (ATCC 10102) with an MBC value of 2 mg/mL. However, there were no MBC values recorded against *P. aeruginosa* (ATCC 27853) and *E. coli* (ATCC 25922) within the utilized concentrations (Table 3). The extract was further evaluated for its bactericidal effect using MBC/MIC ratios. It displayed the MBC/MIC value of 4 on both Gram-positive strains (Table 3).

### Effect of the extract on RCD activity

**Fig. 1:**
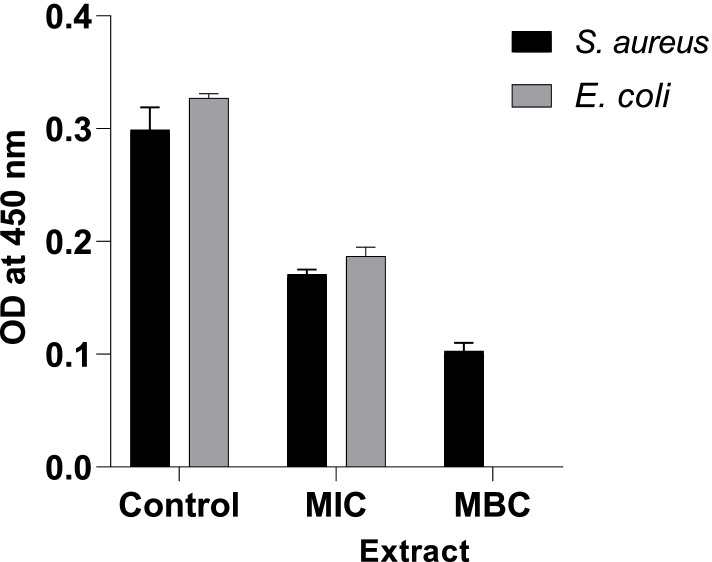
Effects of the extract on the RCD activity of *S. aureus* and *E. coli*

The inhibitory effect of the extract against RCD activity was evaluated by a spectrophotometric assay based on the reduction of iodonitrotetrazolium chloride (INT) by the respiratory chain dehydrogenase. When the MIC and MBC were added against *S. aureus* (ATCC 25925), the absorbance value significantly decreased to 0.171 and 0.103, respectively, compared with the control (0.299). It was also noted that the absorbance value of *S. aureus* (ATCC 25925) treated with the MIC was higher in contrast with that of the MBC (Fig. 1). Moreover, the absorbance also decreased when *E. coli* (ATCC 25922) was treated with the MIC (0.187) in comparison to the control value (0.327).

### Characterization of the bacterial extract

#### Functional groups of the extract

**Fig. 2:**
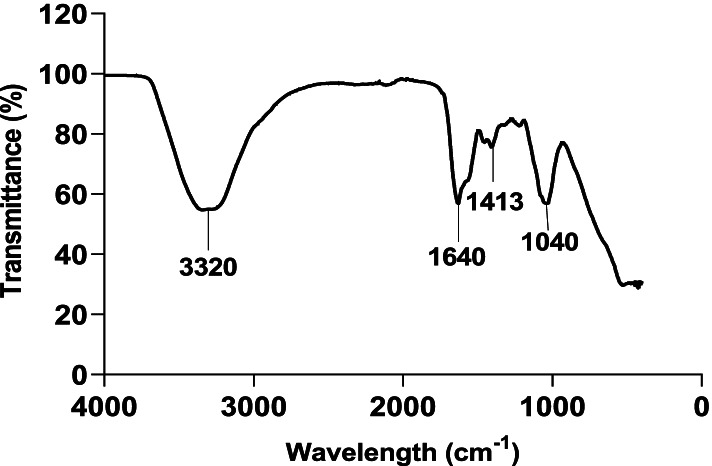
FTIR spectrum of the extract from endophytic *S. olivaceus* CP016795.1

FTIR spectroscopy is recognized as an important tool for the identification of functional groups within the bioactive components. FTIR spectrum displayed a strong and broad absorption peak at 3320 cm^− 1^, as a result of O-H stretching or N-H stretching vibrations, representing alcohol and/or amine groups (Fig. 2). The extract also revealed a strong absorption peak at 1640 cm^− 1^, due to the C=O stretching, showing an amide group. There was a medium absorption at 1413 cm^− 1^, as a result of C=C stretching vibration, representing an aromatic group. Lastly, the strong and broad absorption peak was observed at 1040 cm^− 1^, due to medium C-N stretching vibration, indicating the presence of an amine group.

### Classes of the chemical constituents of the extract

A qualitative evaluation of different classes of compounds within the bacterial extract was conducted. The compounds: phenols, flavonoids, quinonis, and coumorins were present whereas saponins and anthraquiones were not detected (Table 4).

**Table 4:** The qualitative chemical constituents of the bacterial extract

Compounds	Presence of compounds
Phenols	+
Flavonoids	+
Quinones	+
Coumarins	+
Anthraquinones	–
Saponins	–

Key: + indicates presence and – denotes absence

### Volatile compounds of the extract

GC-MC chromatogram profile of the extracted secondary metabolites revealed a total of 192 compounds (Table 5, Table S1). The 5 main compounds were 5-hydroxymethylfurfural (19.47%), 4H-pyran-4-one,2,3-dihydro-3,5-dihydroxy-6-m (10.51%), 2-(1-hydroxyethyl)-2-methyl-1,3-oxathiolane (7.11), 1-nitro-1-deoxy-d-glycero-l-mannoheptitol (5.80%) and 2H-pyran-2-acetic acid, tetrahydro (3.67%).

**Table 5:** Predominant chemical constituents of the extracted secondary metabolites identified by GC-MS.

Peak	Compounds	Area%
1	5-Hydroxymethylfurfural	19.47
2	4H-Pyran-4-one, 2,3-dihydro-3,5-dihydroxy-6-methyl	10.51
3	2-(1-Hydroxyethyl)-2-methyl-1,3-oxathiolane	7.11
4	1-Nitro-1-deoxy-d-glycero-l-mannoheptitol	5.80
5	2H-Pyran-2-acetic acid, tetrahydro	3.67

## Discussion

Endophytic actinomycetes present a relatively under-explored reservoir of pharmacologically active compounds [32]. They are known to produce specific chemical entities with special structures of medicinal importance. Their compounds have been implicated in several pharmacological activities such as antioxidants, and antimicrobial actions among others [33]. Thus, in this study, endophytic actinobacterium associated with *A. ferox* Mill was isolated and screened for the production of antibacterial compounds.

Surface sterilization of the aloe tissues was done to eliminate epiphytes. The control plates did not show any bacterial or fungal growth after 48 hours. This meant that the surface sterility assay was successful at inhibiting epiphytes. Thus, the subsequent isolates were true endophytic actinomycetes. The 11 isolates were actinomycetes as illustrated by the phenotypical characteristics of colour and pigment production. The results were in agreement with previous findings [34].

The ISO01 strain exhibited better antibacterial activity in comparison with the other strains during primary and secondary antibiotic production screening. The difference might be due to the differences in the production of secondary metabolites with antibacterial effects. Literature states that approximately 43.4% of endophytic actinobacteria obtained from leaves and roots display antibacterial activity [35–37]. Therefore, due to financial constraints, only ISO01 was identified using I6S rRNA gene sequence analysis. The actonobacterium was confirmed to be *S. olivaceus* CP016795.1. *Streptomyces olivaceus* has been reported to produce cholesterol oxidase [38], anti-leukemic glutaminase free L-Asparaginase [39], endo-β-1,4-xylanase, used in the bleaching process [40], and cellulase with ability to produce bioethanol from cellulolytic agro-residues [41]. To our knowledge, this actinobacterium has not been recorded to produce antibacterial compounds. However, the results are consistent with various reports stating that the most abundant isolates of the endophytic actinomycetes belong to the genus *Streptomyces* [42, 43].

Secondary metabolites are synthesised genes located in continuous DNA segments known as biosynthetic secondary metabolite gene clusters [44]. Traditionally, biosynthetic gene clusters include non-ribosomal peptide synthase (NRPS), N-**γ**-acetylglutaminylglutamine1-amide (NAGGN), and polyketide synthase (PKS) family clusters [45, 46]. Different gene clusters often encode for different types of secondary metabolites. Approximately 48% of the revealed biosynthetic gene clusters showed more or equal to 50% (50 ≤ x) percentage similarity, implicating the accurate prediction of the secondary metabolites produced by *S. olivaceus* CP016795.1. The actinobacterium was assumed to certainly synthesise germicidin, ectoine, albaflavenone, phenalinolactone A, hopene, and geosmin as they demonstrated above 90% similarity to the gene clusters responsible for the production of these metabolites [47]. Moreover, the high number of the revealed biosynthetic genes implies the ability and potential of this actinobacterium to produce diverse metabolites of biotechnological and medicinal importance. For example, PKS, NRPS, RiPPs (ribosomally synthesised and post-translationally modified peptides), terpenes, and thiopeptides families are best known to produce potent inhibitors of bacterial growth [48–50]. Siderophores inhibit microbial growth by scavenging iron from biomolecules [51].

The extract illustrated a promising antibacterial effect against the tested strains. However, there were no MBC values recorded against *P. aeruginosa* (ATCC 27853) and *E. coli* (ATCC 25922) within the utilized concentrations. Thus, the extract was considered to have bactericidal action only against the Gram-positive strains and not the Gram-negative. When the ratio MBC/MIC is ≤4, extracts are considered to have a bactericidal effect and when the MBC/MIC ratio is > 4, their effect is defined as bacteriostatic [52] Thus, the extract was further affirmed to possess bactericidal ability against both Gram-positive *strains, S. aureus* (ATCC 25925) and *B. cereus* (ATCC 10102). The difference in the activity of the extract against different classes of bacteria was attributed to the differences in the peptidoglycan layer of the outer membrane. The thinner outer membrane layer of Gram-negative bacteria in comparison to the Gram-positive bacteria consists of an additional protective lipopolysaccharide layer that exhibits resistance and antigenicity against antimicrobials [53]. Therefore, a conclusion was drawn that the slight resistance of Gram-negative bacteria *E. coli* (ATCC 25922) in comparison to the Gram-positives (ATCC 25925) was due to the lipopolysaccharide layer [54]. Nigussie et al. [55] observed similar results whereby the extract’s antibacterial activity was more profound against the Gram-positive bacteria than the Gram-negative strains. Thus, the results imply that the bacterial extract has the potential to be utilised as the source of therapeutic antibacterial agents, especially against Gram-positive bacteria.

The inhibitory effect of the extract against RCD activity was evaluated by a spectrophotometric assay based on the reduction of iodonitrotetrazolium chloride (INT) by the respiratory chain dehydrogenase [56]. The results demonstrated that the extract has the potential to exert inhibitory activity and killing effect against the tested strains by interfering with the activity of the respiratory chain dehydrogenase. The interference of the bacteria’s respiratory chain dehydrogenase by the extract implies the deactivation of oxidative phosphorylation, which is responsible for bacteria’s energy production [57]. Therefore, the bacteria might be inhibited or killed due to the lack of energy.

The pharmaceutical significance of endophytic actinomycetes lies in the activity of the functional groups of their secondary metabolites. The hydroxyl group is an essential part of most phenolic composites such as flavonoids [58]. According to Burman et al. [59], flavonoids possess antibacterial and antifungal properties. Therefore, the FT-IR results provide further supporting proof for the presence of phenolic compounds in the bacterial extract. Moreover, the varied functional groups also suggest the presence of dissimilar classes of compounds (phenols, flavonoids, quinones, and coumarins) with potential antibacterial activities [19, 60]. The GC-MS analysis also revealed a high number of compounds. The high number of the detected compounds may be due to the high number of the identified biosynthetic secondary metabolites gene clusters (see Table 2). Thus, the antibacterial activity of the extract may be assigned to the existence of many bioactive compounds, which have been reported to have antimicrobial activity, such as 5-hydroxymethylfurfural, 4H-pyran-4-one, 2,3-dihydro-3,5-dihydroxy-6-methyl, tetradecanoic acid, cis-vaccenic acid and trans-2-dodecenoic acid [20, 53, 55, 61–63]. The antibacterial efficacy of the extracted metabolites may also be due to the synergistic effect of the different identified compounds [64]. Therefore, the *S. olivaceus* CP016795.1 has the potential to serve as a source of effective antibacterial compounds.

## Conclusions

ISO1-*S. olivaceus* CP016795.1 demonstrated the capability to produce antimicrobial compounds. Its secondary metabolites displayed noteworthy inhibitory effects (MIC ≤1 mg/mL) against *S. aureus* (ATCC 25925) and *E. coli* (ATCC 25922. The extract gave the evidence to exert antibacterial activity by interfering with the respiratory chain dehydrogenase’s activity. The extract revealed the presence of different classes of compounds and functional groups that were perceived to have contributed to its potent antibacterial activity. Thus, *S. olivaceus* CP016795.1 has the potential to serve as a source of compounds of pharmacological importance. Further studies are imperial to pinpoint the different mechanisms the revealed bioactive compounds exert during their antibacterial activity.

## Supplementary Information

**Additional file 1: Table S1.** Chemical constituents of the extracted secondary metabolites identified by GC-MS.

## Data Availability

The bacterium analysed in this study is available from Genbank (https://www.ncbi.nlm.nih.gov/genbank/). The bacterial accession number is provided in the manuscript. The other datasets used during the current study are available from the corresponding author on reasonable request.

## Abbreviations

*A. ferox* Mill: *Aloe ferox* Mill
*S. aureus*: *Staphylococcus aureus*
*B. cereus*: *Bacillus cereus*
*E. coli*: *Escherichia coli*
*S. olivaceus*: *Streptomyces olivaceus*
AIAG: actinomycete isolation agar with glycerol
NA: nutrient agar
MH: Mueller Hinton
CFU: colony forming units
NCBI: National Center for Biotechnology Information
MIC: minimum inhibitory concentration
MBC: minimum bactericidal concentration
ATCC: American Type Culture Collection
DMSO: dimethyl sulfoxide
INT: piodonitrotetrazodium violet
BLAST: basic local alignment search tool
NRPS: non-ribosomal peptide synthase
NAGGN: N-γ-acetylglutaminylglutamine1-amide
PKS: polyketide synthase
T3PKS: type III polyketide synthase
T1PKS: type I polyketide synthases
LAP: Linear azol(in)e-containing peptide
RCD: respiratory chain dehydrogenase
FTIR: Fourier transform-infrared spectrophotometer
GC-MS: gas chromatography-mass spectrophotometry
