# Supplementary material for: Bioprospecting of endophytic actinobacterium associated with Aloe ferox mill for antibacterial activity
Source: BMC Complement Med Ther. 2022 Oct 3;22:258. doi: 10.1186/s12906-022-03733-8 (PMC9531469; doi:10.1186/s12906-022-03733-8)
Supplement: Supplementary file 1 — Additional file 1: Table S1. Chemical constituents of the extracted secondary metabolites identified by GC-MS. [file 12906_2022_3733_MOESM1_ESM.docx]

**Table S1.** Chemical constituents of the extracted secondary metabolites identified by GC-MS

| **Peak** | **Compounds** | **Area%** | **Peak** | **Compounds** | **Area%** | **Peak** | **Compounds** | **Area%** |
| --- | --- | --- | --- | --- | --- | --- | --- | --- |
| 1 | Methyl Isobutyl Ketone | 0.09 | 12 | 4-sec-Butoxy-2-butanone | 0.24 | 23 | Methylphoshonic acid | 0.05 |
| 2 | 1-Butanol, 2-methyl-, (S) | 0.17 | 13 | Beta.-Ethoxypropionic acid | 0.23 | 24 | 2,4-Dimethyl-5-methylthiopent-4-en-2-o | 0.40 |
| 3 | Pentane, 1-ethoxy | 0.12 | 14 | Acetate | 0.04 | 25 | Cyclohexane, (2-methylpropyl) | 0.49 |
| 4 | 1-Butanol, 3-methyl | 0.21 | 15 | 1,4,2,5 Cyclohexanetetrol | 0.04 | 26 | α-L-lyxo-Hexopyranoside, methyl 3-  amino | 0.11 |
| 5 | 2,4,6-Trimethyl-1-nonene | 0.18 | 15 | p-Xylene | 0.29 | 27 | 2,5,6-Trimethyl-1,3-oxathiane | 0.85 |
| 6 | Phospholane | 0.11 | 17 | 1,1-Cyclohexanedimethanol | 0.41 | 28 | d-Glycero-d-galacto-heptose | 0.08 |
| 7 | 3-Isopropoxy alanine | 0.20 | 18 | 1,5-Heptadiene, 2-methyl- | 0.17 | 29 | D-Mannoheptulose | 0.23 |
| 8 | 1-Butanol, 2-nitro | 0.88 | 19 | 4-Aminocyclohexanone, N-acetyl | 0.06 | 30 | 2-Hydroxymethyl-9-[.beta.-d-ribo-  furanosyl]hyp | 0.16 |
| 9 | Butanoic acid, ethyl ester | 0.59 | 20 | 2-Furanmethanol | 0.39 | 31 | 1R,3-cis-Diethoxy-5-trans-methylcyclo-  hexane | 0.04 |
| 10 | Propane, 1,1-diethoxy | 0.94 | 21 | Bicyclo[2.1.1]hexan-2-ol, 2-ethenyl | 0.14 | 32 | 1,3-Dimethyl-4,8-dioxatricy-  clo[5.1.0.0(3,5)]octane-2,6-diol | 0.03 |
| 11 | Propane, 1-(1-ethoxyeth-  oxy) | 0.61 | 22 | 6-Acetyl-.beta.-d-mannose | 0.01 | 33 | 2-Furancarboxaldehyde, 5-methyl | 0.11 |

**Table S1.** (Continued)

| **Peak** | **Compounds** | **Area%** | **Peak** | **Compounds** | **Area%** | **Peak** | **Compounds** | **Area%** |
| --- | --- | --- | --- | --- | --- | --- | --- | --- |
| 34 | 2-t-Butyl-5-propyl-[1,3]dioxolan-4-one | 0.42 | 45 | Cyclohexanamine, N-3-butenyl-N-methyl | 1.21 | 56 | Decane, 3,6-dimethyl | 0.03 |
| 35 | Butanedioic acid, dimethyl ester | 0.09 | 46 | Thymine | 1.06 | 57 | Undecane, 4,7-dimethyl | 0.44 |
| 36 | Octane, 2,3,6,7-tetramethyl | 0.09 | 47 | 3-Acetylthymine | 0.79 | 58 | 2-(2-Ethoxyethoxy)ethyl 2-methyl- butanoate | 1.26 |
| 37 | Heptane, 3,3,5-trimethyl | 0.02 | 48 | Spiro[tetrahydrofuran-3,5'-hydantoin] | 1.22 | 59 | Desulphosinigrin | 0.44 |
| 38 | Butanedioic acid | 0.36 | 49 | Butanedioic acid, hydroxy-, dimethyl es- ter | 0.62 | 60 | 2,4:3,5-Dimethylene-l-iditol | 0.28 |
| 39 | S-[2-Aminoethyl]-dl-cysteine | 0.08 | 50 | Propanoic acid, 3-(acetyloxy)-2-(hy-  droxymethyl | 0.27 | 61 | Propionic acid, 3-mercapto-, isooctyl  ester | 0.22 |
| 40 | Cyclohexylmethyl S-2-(diisopropyla-  mino) ethyl | 0.09 | 51 | Formic acid, octyl ester | 0.04 | 62 | 1R,3-trans-Dimethoxy-2-methylcy-  clohexane | 0.17 |
| 41 | Ethyl 5-methylhexanoate | 0.05 | 52 | 1-Butanol, 3-methyl, acetate | 0.32 | 63 | 5-Hydroxymethylfurfural | 19.47 |
| 42 | 2,5-Dimethyl-4-hydroxy-3(2H)- furanone | 0.10 | 53 | 4H-Pyran-4-one, 2,3-dihydro-3,5-dihy- droxy-6-methyl | 10.51 | 64 | 2-(1-Hydroxyethyl)-2-methyl-1,3-ox- athiolane | 7.11 |
| 43 | 4-Fluoro-1-methyl-5-carboxylic acid | 0.03 | 54 | Fluoroacetic acid, dodecyl ester | 0.05 | 65 | 4-Hepten-3-one, 5-methyl | 0.61 |
| 44 | Undecane | 0.16 | 55 | Decane, 4-ethyl | 0.02 | 66 | Tetradecane, 1-fluoro | 0.74 |

**Table S1.** (Continued)

| **Peak** | **Compounds** | **Area**  **%** | **Peak** | **Compounds** | **Area**  **%** | **Peak** | **Compounds** | **Area**  **%** |
| --- | --- | --- | --- | --- | --- | --- | --- | --- |
| 67 | 2-Thiopheneacetic acid, 2-tridecyl ester | 0.52 | 77 | 5-Amino-1-pentanol, trimethylsilyl ether | 0.82 | 87 | Succinic acid, 2-ethylhexyl 2-methyl-3- pentyl ester | 0.26 |
| 68 | α-D-Glucopyranose, 2-amino-3,6-anhydro | 1.24 | 78 | D-Fructose, 1,3,6-trideoxy-3,6-epithio | 0.18 | 88 | Furane-2-carboxylic acid, 5-(4-chloro-3-  methylphenoxymethyl) | 0.24 |
| 69 | 1-Nitro-1-deoxy-d-glycero-l-mannoheptitol | 5.80 | 79 | Nonahexacontanoic acid | 0.31 | 89 | Nonane, 5-(1-methylpropyl) | 0.05 |
| 70 | 2H-Pyran-2-acetic acid, tetrahydro | 3.67 | 80 | Thiirane, octyl | 0.10 | 90 | Dodecane, 2,6,11-trimethyl | 0.14 |
| 71 | 9-Acetoxynonanal | 0.17 | 81 | 5-Methyl-1R,3-trans-cyclohexanediol | 0.24 | 91 | Succinic acid, heptyl 2-methylpent-3-yl  ester | 0.26 |
| 72 | Tertbutyloxyformamide, N-methyl-N-[4-(1-pyr-  rolidinyl)-2-butynyl] | 0.38 | 82 | Acetic acid, 3,7,11,15-tetramethyl-hexa-  decyl ester | 0.11 | 92 | Succinic acid,2,4-dimethylpent-3-yl  nonyl ester | 0.09 |
| 73 | Thiazole, 2-ethoxy | 0.85 | 83 | 2-(2-Vinyloxy-ethoxy)-cyclohexanol | 0.05 | 93 | Heptanoic acid, 3-oxo-, methyl ester | 0.50 |
| 74 | Valeric acid, tetradecyl ester | 1.30 | 84 | 3-Cyclopentylpropionic acid, 2-tetrahy-  drofurylmethyl ester | 0.57 | 94 | 7-[β-d-Ribofuranosyl]imidazo[4,5-  d][1,2,3]Methyl beta.-d-ribofuranoside | 0.36 |
| 75 | Tetradecane | 0.32 | 85 | Acetic acid, 1-(2-hydroxy-1-methyl-  ethyl)-3-methoxymethoxy-2-methyl- propyl ester | 0.06 | 95 | Phenol, 2,5-bis(1,1-dimethylethyl) | 0.32 |
| 76 | 2-Thiazoleamine, 4-(tetrahydro-2-oxofuran-5-  yl) | 0.07 | 86 | exo-1,2-O-Ethylidene-.alpha.-d-  erythrofuranose | 0.13 | 96 | Methyl.β-d-ribofuranoside | 0.11 |

| **Peak** | **Compounds** | **Area%** | **Peak** | **Compounds** | **Area%** | **Peak** | **Compounds** | **Area%** |
| --- | --- | --- | --- | --- | --- | --- | --- | --- |
| 97 | D-Fructose, 1,3,6-trideoxy-3,6-epithio | 0.57 | 108 | 5-Keto-2,2-dimethylheptanoic acid, ethyl(ester) | 0.16 | 119 | Dodecane, 1-fluoro | 0.04 |
| 98 | 3-Pentanol, 2,2,4,4-tetramethyl-3-(tetra-  hydo-2-furyl) | 0.51 | 109 | Diethylmalonic acid, monochloride,  4-octyl ester | 0.06 | 120 | d-Glucitol, 1-thio-nonyl | 0.05 |
| 99 | Cyclohexane, 1R-acetamido-4cis-ace-  toxy-5,6Zc | 0.09 | 110 | Tetradecyl trifluoroacetate | 0.69 | 121 | Glycine, N-isobutoxycarbonyl-, octadecyl  ester | 0.07 |
| 100 | 1,2,4-Trioxolane-2-octanoic acid, 5-oc-  tyl-, methyl ester | 0.15 | 111 | Hexadecane | 0.10 | 122 | D-Galactose, diethyl mercaptal, pentaacetate | 0.36 |
| 101 | R-(+)-Methyl-2-isopropyl-5-oxohexano-  ate | 0.05 | 112 | 2-[2-Hydroxyethyl]-9-[.beta.-d-ribo-  furanosyl] hypoxanthine | 0.07 | 123 | 4-Dimethylsilyloxytridecane | 0.21 |
| 102 | Furazan-3-carbohydrazide, 4-amino | 0.17 | 113 | l-Gala-l-ido-octose | 0.05 | 124 | Methyl 2,6-anhydro-.alpha.-d-altroside | 0.07 |
| 103 | Nonanoic acid | 0.74 | 114 | 6-Oxododecanedioic acid | 0.06 | 125 | Methane, [(hydroxy)bis(trifluorome- thyl)borinato](dimethylammoniato)trime- thylsilyl) | 0.24 |
| 104 | Formic acid, dec-2-yl ester | 0.29 | 115 | Hexanedioic acid, mono(2-  ethylhexyl) ester | 0.05 | 126 | Hexanoic acid, pentadecyl ester | 0.18 |
| 105 | Tetraethylenepentamine | 0.15 | 116 | n-Propyl nonyl ether | 0.05 | 127 | Thiofanox | 0.07 |
| 106 | Methyl 6-O-[1-methylpropyl]-.beta.-d-  galactopyranoside | 0.16 | 117 | 1-Undecanol, acetate | 0.02 | 128 | Decanoic acid, 10-fluoro-, trimethylsilyl es-  ter | 0.35 |
| 107 | 1H-Xanthine, 3,7-dimethyl-1-(3-trime-  thylsilylpropyl) | 0.05 | 118 | 3,4-Difluorobenzoic acid, 2-tridecyl  ester | 0.03 | 129 | d-Glycero-l-gluco-heptose | 0.10 |

**Table S1.** (Continued)

**Table S1.** (Continued)

| **Peak** | **Compounds** | **Area%** | **Peak** | **Compounds** | **Area%** | **Peak** | **Compounds** | **Area%** |
| --- | --- | --- | --- | --- | --- | --- | --- | --- |
| 130 | Eicosane | 0.58 | 141 | Octaethylene glycol monododecyl ether | 0.75 | 152 | (3,4-Dimethyl-5-oxodihydrofuran-2-ylidene) acetic acid, t-butyl ester | 0.01 |
| 131 | β-l-Rhamnofuranoside, methyl-5-O-  acetyl | 0.47 | 142 | 5-Amino-1-pentanol,  trimethylsilyl ether | 0.14 | 153 | n-Propyl nonyl ether | 0.04 |
| 132 | Cyclopropane-1-carbohydrazide, 2-  phenyl-N2-(3-methylcyclohexylideno) | 1.06 | 143 | 1-[N-Aziridyl]propane-2-thio | 0.15 | 154 | α-D-Glucopyranoside, methyl 4,6-O-  nonylidene | 0.02 |
| 133 | cis-2,5-Dimethylthiane | 0.42 | 144 | (Z)-4-Decen-1-ol, trimethylsilyl  ester | 0.05 | 155 | 1,1-Dimethyl-3-(3-methyl-1,2,4-oxadiazol-5-  yl) | 0.02 |
| 134 | N-Nitroso-2-methylthiazolidine | 0.81 | 145 | Tetradecanoic acid | 2.58 | 156 | 3-Chloropropionic acid, heptadecyl ester | 0.09 |
| 135 | 3-Deoxy-d-mannoic lactone | 1.49 | 146 | 7-Octadecanone | 0.02 | 151 | 1-Di(tert-butyl)silyloxyhexadecane | 0.08 |
| 136 | i-Propyl 5,9-hexacosadienoate | 0.17 | 147 | β-d-Mannofuranoside, methyl-  2,3-O-(ethylboranediyl) | 0.02 | 158 | Octadecanoic acid, 2-hydroxy, methyl ester | 0.13 |
| 137 | 8-Octadecanone | 0.33 | 148 | Tetradecyl trifluoroacetate | 0.42 | 159 | Fumaric acid, allyl undecyl ester | 0.03 |
| 138 | Pentane, 1,1-bis(2-thiazolylamino) | 0.80 | 149 | Pentanoic acid, octyl ester | 0.02 | 160 | 7,9-Di-tert-butyl-1-oxaspiro(4,5)deca-6,9-  diene-2,8-dione | 0.08 |
| 139 | 2-Methylhexacosane | 0.52 | 150 | Pentadecanoic acid | 0.07 | 161 | Pentadecanoic acid, 14-methyl-, methyl ester | 0.24 |
| 140 | 3,4:5,6-Di-O-isopropylidene-2-deoxy-2-  acetamido-d-glucitol | 0.09 | 151 | 3-(1,3-Dihydroxyisopropyl)-  1,5,8,11-tetraoxacyclotridecane | 0.01 | 162 | l-(+)-Ascorbic acid 2,6-dihexadecanoate | 0.15 |

**Table S1.** (Continued)

| **Peak** | **Compounds** | **Area**  **%** | **Peak** | **Compounds** | **Area%** | **Peak** | **Compounds** | **Area**  **%** |
| --- | --- | --- | --- | --- | --- | --- | --- | --- |
| 163 | Benzenepropanoic acid, 3,5-bis(1,1-di- methylethyl)-4-hydroxy-, methyl ester | 0.11 | 174 | 2-Undecenal, E | 0.14 | 185 | 12,14,14-Trimethyl-3,6,9-tri- oxapentadecan-1-ol | 0.01 |
| 164 | Erucic acid | 0.31 | 175 | Heptadecanoic acid | 0.11 | 186 | 11-Methyloctadec-12-enoic acid,  methyl ester | 0.09 |
| 165 | cis-5-Dodecenoic acid, dimethyl(3,3,3-  trifluoro | 0.01 | 176 | (Z)-3,7,11-Trimethyldodec-2-enoic acid,  methyl | 0.02 | 187 | 9,12-Octadecadienoic acid (Z,Z) | 0.34 |
| 166 | Cyclohexanone, 6-methyl-3-(1-meth-  ylethyl)-2-(2-oxopropyl) | 0.06 | 177 | Eicosanoic acid | 0.80 | 188 | Oleic acid, 3-hydroxypropyl ester | 0.08 |
| 167 | Pentadecanoic acid | 1.84 | 178 | Undecanoic acid, 11-mercapto | 0.08 | 189 | cis-Vaccenic acid | 0.38 |
| 168 | trans-7-Carbomethoxy-2-octenedioic acid, dimethyl ester | 0.00 | 179 | 1-Fluorononane | 0.03 | 190 | Octadecanoic acid | 0.34 |
| 169 | 1,7-Dioxa-10-thia-4,13-diazacyclopenta-  deca-5 | 0.01 | 180 | 4,6-Di-O-acetyl-1,5-anhydro-2,3-di-O-me-  thyl-D | 0.01 | 191 | (Z)-4-Decen-1-ol, trimethylsilyl es-  ter | 0.03 |
| 170 | 6-Amino-1-hexanol, trimethylsilyl ether | 0.02 | 181 | Cyclopentanecarboxylic acid, dodec-9-ynyl  ester | 0.07 | 192 | 2-Trifluoroacetoxypentadecane | 0.44 |
| 171 | 1-Nonadecene | 0.20 | 182 | 6-Octadecenoic acid | 0.11 |  |  |  |
| 172 | Hexadecanoic acid, 14-methyl-, methyl ester | 0.12 | 183 | 2,3-Diazabicyclo[3.2.0]hept-2-ene, 1,6,6,-tri- fluoro-4-spirocyclopropane | 0.02 |  |  |  |
| 173 | trans-2-Dodecenoic acid | 0.06 | 184 | 1-Methyl-1-(4-methylpentyl)oxy-1-silacy-  clobutane | 0.04 |  |  |  |
